# Genetic Susceptibility and Mechanisms Underlying the Pathogenesis of Anthracycline-Associated Cardiotoxicity

**DOI:** 10.1155/2022/5818612

**Published:** 2022-08-03

**Authors:** Yonghe Ding, Ke Du, Yu-Juan Niu, Yong Wang, Xiaolei Xu

**Affiliations:** ^1^The Biomedical Sciences Institute of Qingdao University Qingdao (Branch of SJTU Bio-X Institutes), Qingdao University, Qingdao 266021, China; ^2^Department of Biochemistry and Molecular Biology, Department of Cardiovascular Medicine, Mayo Clinic, Rochester, MN 55902, USA; ^3^School of Public Health, Qingdao University, Qingdao 266021, China; ^4^School of Traditional Chinese Medicine, Beijing University of Chinese Medicine, Beijing 10029, China

## Abstract

Anthracyclines are chemotherapeutic agents widely used to treat a variety of cancers, and these drugs have revolutionized our management of cancer patients. The dose-dependent cardiotoxicity of anthracyclines, however, remains one of the leading causes of chemotherapy treatment-associated mortality in cancer survivors. Patient threshold doses leading to anthracycline-induced cardiotoxicity (AIC) are highly variable among affected patients. This variability is largely ascribed to genetic variants in individuals' genomes. Here, we briefly discuss the prevailing mechanisms underlying the pathogenesis of AIC, and then, we review the genetic variants, mostly identified through human genetic approaches and identified in cancer survivors. The identification of all genetic susceptibilities and elucidation of underlying mechanisms of AIC can help improve upfront risk prediction assessment for potentially severe cardiotoxicity disease and provide valuable insights into the understanding of AIC pathophysiology, which can be further leveraged to develop targeted pharmacogenetic therapies for those at high risk.

## 1. Introduction

Anthracyclines are one of the most widely used chemotherapy drugs for treating a variety of hematologic malignancies and solid tumors in both pediatric and adult patients. Since their first introduction in the 1960s, anthracyclines have been among the most potent antineoplastic therapeutics available in clinical practice to date [1, 2]. Some anthracycline drugs, such as doxorubicin, are also listed as essential cytotoxic medicines by the World Health Organization [3]. It was estimated that approximately 50-60% of cancer survivors had been treated with at least one anthracycline class of drugs [4, 5]. Owing to the contribution of anthracyclines and other improvements in cancer treatment, the 5-year survival rates for both pediatric and adult cancer patients have increased dramatically [6]. However, anthracycline-induced cardiotoxicity (AIC) side effects, ranging from asymptomatic cardiac function decline to heart failure and even death, now represent a major cause of treatment-related morbidity and mortality in a considerable number of cancer survivors. AIC can be traditionally classified into 3 distinct phases including acute, subchronic (early onset), and chronic (late onset), respectively [1, 7, 8]. Acute AIC usually occurs during the course of or within 1 week after anthracycline therapy, manifesting mostly transient sinus tachycardia and arrhythmias which are relatively rare and usually reversible. Subchronic AIC is defined to occur within 1 year after completion of anthracycline therapy, which represents the most common form of AIC manifesting irreversible myocarditis and left ventricular dysfunction, whereas chronic AIC occurs more than 1 year or even decades after cessation of the anthracycline treatment, featuring irreversible cardiomyopathy, arrhythmia, and eventual heart failure which is a major clinical concern. In modern era, the estimated incidence of cardiac dysfunction and heart failure in cancer survivors who received anthracycline treatment can reach up to 9% [9, 10].

High cumulative doses are a well-established risk factor for AIC. For example, one study showed that among cancer survivors who received an anthracycline cumulative dose of >300 mg/m^2^, ~28% manifested cardiac dysfunction and heart failure, compared to ~7% among those who received cumulative doses of <250 mg/m^2^ [11–14]. Other risk factors for AIC include age, radiation therapy, and preexisting conditions such as diabetes, hypertension, coronary artery disease, and other cardiovascular risk factors like obesity, smoking, and hyperlipidemia [15–19]. In addition to these nongenetic risk factors, it is also clear that genetic variants contribute significantly to the incidence and severity of AIC among affected patients. For example, for a given dose, some patients experience no overt cardiac dysfunction and/or other AIC effects, while others in extreme cases manifest severe congestive heart failure and/or even death [20]. Quite often, some patients receive high doses of anthracycline treatment (>500 mg/m^2^) without incurring any overt AIC, while others experience AIC even at doses of <200 mg/m^2^ [13]. These large variabilities are largely ascribed to genetic variants in interindividual genomes. Identifying and elucidating all the genetic susceptibilities to AIC can help improve upfront risk assessment for potential severe cardiotoxicity disease prediction and provide valuable insights into the understanding of disease pathophysiology, which can be further leveraged to develop targeted pharmacogenetic therapies for those at high risk.

**Figure 1:**
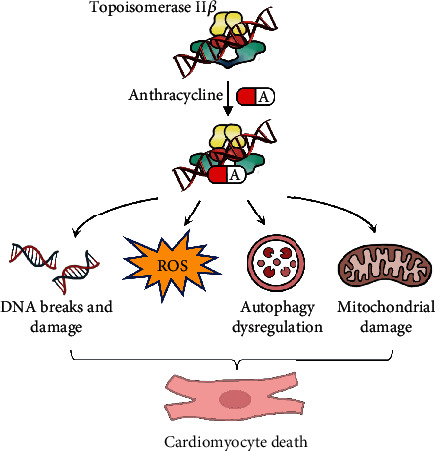
Pathogenic mechanisms of anthracycline-induced cardiotoxicity.

**Figure 2:**
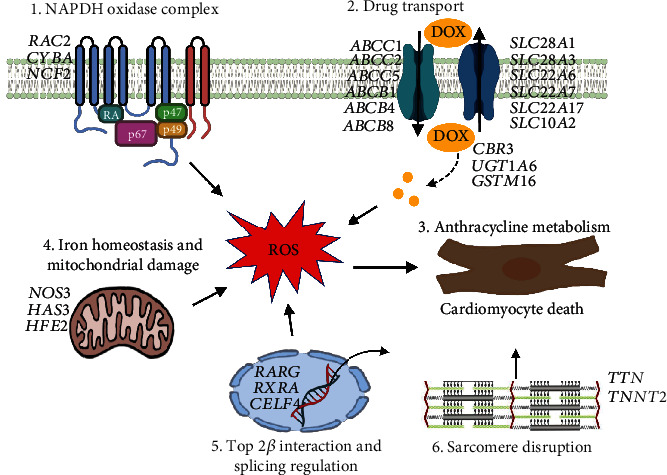
Summary of key affected genes with variants identified to be associated with anthracycline-induced cardiomyocyte toxicity categorized based on the proposed mechanisms of action. DOX: doxorubicin; ROS: reactive oxygen species.

To identify genetic variants associated with AIC, human genetic studies, mostly including both candidate gene approaches and genome-wide association studies (GWASs), have been employed in both pediatric and adult cancer survivors [4]. Both approaches have yielded more than 60 significant single nucleotide polymorphisms (SNPs) in approximately 40 affected genes [4, 5, 21]. Notably, most of the human genetic studies aiming to identify variants associated with AIC reported in the literature are limited by relatively small sample sizes and a lack of independent replication and functional validation [4, 5, 22, 23]. Here, in this brief review, we first discuss the well-studied and emerging mechanisms underlying the pathogenesis of AIC (Figure 1). We then review key genetic variants associated with AIC, which are categorized based on the action of mechanisms with the corresponding affected genes if available, as summarized Figure 2. Last, we propose human-induced pluripotent stem cell cardiomyocytes (hiPSC-CMs) and zebrafish as prolific in vitro and in vivo models, respectively, to perform functional validation analyses of the variants of interest to better understand the genetic mechanisms of AIC.

## 2. Pathogenic Mechanisms of AIC

Despite active research over many decades, the pathogenic mechanisms of AIC remain incompletely understood. Initially, one of the prevailing and well-accepted hypotheses for AIC was related to the generation of excess reactive oxygen species (ROS) during the process of anthracycline drug metabolism, in which electron exchange between the anthracycline quinone moiety and oxygen molecules is the major direct cause of cardiomyocyte death and AIC. Because cardiomyocytes contain a high volume of mitochondria, which rely heavily on the metabolism of oxidative substrates [24], further promoted by the fact that the heart has limited ability to clear ROS generated by various scavengers, cardiomyocytes are especially highly sensitive to oxidative stress. In addition, oxygen radicals can also be generated by complexes formed from anthracycline and iron that undergo redox cycling [25, 26], which can further deteriorate the AIC effect. Dexrazoxane, a derivative of ethylenediaminetetraacetic acid, is an iron chelator that can block iron-assisted generation of oxidative radical. Dexrazoxane was thus developed as the only mechanism-based drug approved by the Food and Drug Administration (FDA) to treat AIC in patients who received anthracycline-based chemotherapy [27]. Nevertheless, depending on different preclinical animal models, the potential benefits of other antioxidants in protecting against AIC are still uncertain [28]. The attempts to develop antioxidant-based therapeutic strategies in clinical settings have only yielded limited efficacy [29–32]. Currently, no other clinically proven effective antioxidants for AIC treatment are available yet, suggesting that other pathogenic mechanisms coexist for AIC.

The other well-recognized mechanism of AIC is related to the inhibition of topoisomerase (Top) 2*β*-mediated DNA double-strand breaks, leading to cardiomyocyte cell death. The major function of the Top2 enzyme is to unwind the DNA strands during DNA replication and transcriptional recombination to prevent cell death. The unique biochemical structure of anthracycline enables it to bind to DNA through intercalation and can stabilize the DNA double strand cut by the Top2 enzyme, thus blocking normal DNA and RNA synthesis, leading to DNA double-strand breaks, which in turn result in p53 activation and subsequent cardiomyocyte cell apoptosis and cell death. There are two isoforms of the Top2 enzyme: Top2*α*, which is found mostly in proliferating cells such as cancer cells, and Top2*β*, which is present predominantly in quiescent cells such as cardiomyocytes. The AIC effect mediated by Top2*β* in cardiomyocytes is believed to be similar to the antitumor effect of anthracycline, by which it disrupts the normal catalytic cycle of cancer cells mediated by the proliferating cancer cells predominantly expressing the Top2*α* isoform, causing DNA double-strand breaks, which ultimately induces cancer cell death [33]. More convincing data to support the Top2*β*-mediated double-stranded DNA damage hypothesis for AIC come from evidence that cardiomyocyte-specific deletion of the Top2*β* enzyme protected model mice from AIC [34, 35].

More recently, stage-dependent dysregulation of autophagy has been proposed to be an important molecular event in the pathogenesis of AIC [36–38]. Autophagy is an evolutionarily conserved cellular recycling process through lysosomal degradation of cytoplasmic organelles and proteins and the recycling of breakdown products in response to a broad range of stressors. Autophagy plays important roles in balancing cell survival and maintenance. In both mouse models and in vitro cell culture systems, anthracycline blocks basal autophagy and autophagic flux by inhibiting lysosome acidification [36]. Mice with haploinsufficiency of the PI3K-associated protein Beclin-1 were protected from AIC in terms of structural and functional changes within the myocardium. Similarly, chemical inhibition of PI3K resulted in enhanced elimination of damaged mitochondria, leading to reduced autophagy initiation, which also conferred cardioprotection from AIC in a mouse model system [37]. Furthermore, our group leveraged an adult zebrafish model to assess the time course of autophagy signaling during AIC disease progression [38]. We found that both the basal autophagy level and autophagy flux were activated in the acute phase of anthracycline treatment, whereas they were downregulated in the late phase of treatment. Cardiac-specific overexpression of *atg7*, a gene encoding the rate-limiting protein critical to the initiation of autophagy in zebrafish, partially restored cardiac dysfunction in the later stages but not in the early stages of AIC. Thus, this study underscores the importance of dynamic autophagy dysregulation as a pathogenic mechanism driving AIC.

In addition, another pathogenic mechanism of AIC is related to mitochondrial damage that has been comprehensively reviewed elsewhere [39–41]. And the mechanisms of hemochromatosis (i.e., the accumulation of iron) and impaired calcium handling due to C-13 alcohol metabolites have also been proposed but have not been extensively studied yet [23, 40, 42].

## 3. Genetic Variants Associated with AIC

While the incidence and severity of AIC are mostly determined by accumulated doses of anthracycline, existing evidence strongly supports the hypothesis that genetic factors also contribute significantly to chemotherapy-induced cardiac dysfunction and heart failure [4, 43]. Notably, combined effects of clinical risk factors and genetic variants would likely further increase the risk to develop AIC in patients who received anthracycline drug chemotherapy [44, 45]. To date, mostly through human genetic studies of both pediatric and adult cancer survivors, variants in approximately 40 affected genes have been identified to be associated with the severity of AIC, with a small portion replicated in independent patient cohorts and/or functionally validated in animal models [4, 5, 21, 22]. Below, we will discuss some key pharmacogenetic variants identified to either increase or reduce the risk of AIC based on the mechanisms of action on the affected genes according to literature reports. These mechanisms include drug transport, anthracycline metabolism, oxidative stress generation, DNA damage, iron homeostasis, and sarcomere dysfunction.

### 3.1. Drug Transport

In humans, there are 49 ATP-binding cassette (*ABC*) genes encoding a large family of transmembrane proteins involved in the transport of a wide range of structurally diverse drug substrates [46]. In the myocardium, ABC transporters mostly export multiple chemotherapeutics, such as anthracyclines, from cardiac cells [47]. At least 8 different variants in 5 different ABC genes, including *ABCC1*, *ABCC2*, *ABCC5*, *ABCB1*, and *ABCB4*, have been identified, mostly through candidate gene analysis approaches, to be associated with AIC in both pediatric and adult cancer patients treated with anthracyclines [48–53]. Thus far, evidence to support the association of this ABC family with AIC is robust, with several variants already validated in replication cohorts. In most cases, variants in these ABC genes often reduce or interfere with the expression of located ABC genes, which can result in drug exporting defects, thus leading to the accumulation of anthracycline in cardiomyocytes and causing an increased risk of cardiac dysfunction and AIC. For example, the transporter encoded by *ABCC1/MRP1*, one of the best studied membrane transporters associated with AIC, is highly expressed in the human myocardium [54]. Reducing the activity of this transporter exacerbates the intracellular accumulation of anthracycline. In contrast, higher *ABCC1* activity is correlated with lower drug levels and reduced cellular toxicity. Additionally, detailed functional study in the mouse animal models revealed that overexpression of *ABCB8*, another ABC family protein encoding gene, facilitated iron export leading to reduced anthracycline drug accumulation and ultimately protected against AIC [55, 56].

The solute carrier (*SLC*) superfamily genes encode transporter proteins that play key roles in the absorption and transportation of amino acids, ions, metals, and fatty acids across cellular membranes. In humans, the SLC superfamily can be further categorized into 65 subfamilies containing 458 transport proteins. Anthracycline is a well-recognized substrate of SLC transporters, through which anthracycline drugs are excreted and renally cleared. Several variants in *SLC* genes have been identified to mostly decrease the risk of AIC. For example, the rs7853758 variant in the *SLC28A3* gene was identified to be associated with AIC resistance, and this finding was replicated in different patient cohorts [57]. Because compelling evidence supports a protective role of *rs7853758* in AIC, pharmacogenomic testing for this variant is recommended for pediatric cancer patients who receive anthracycline chemotherapy [45]. Other variants, such as *rs4982753* in the *SLC22A17* gene, *rs4149178* in the *SLC22A7* gene, *rs487784* in the *SLC28A3* gene, and *rs9514091* in the *SLC10A2* genes, were all identified to have potentially protective effects on AIC [44]. In contrast, only one variant, *rs6591722*, in the *SLC22A6* gene was associated with reduced cardiac function in pediatric cancer patients treated with anthracycline [58].

### 3.2. Anthracycline Metabolism

The cardiotoxic effects of anthracyclines have been attributed to the generation of toxic C-13 alcohol metabolites during anthracycline drug metabolism. Similar to transporter protein-encoding genes, genes involved in anthracycline drug metabolism and clearance can also have a significant impact on the severity of AIC.

The proteins encoded by the carbonyl reductase (*CBR*) gene family are the major enzymes that catalyze many pharmacologic carbonyl compounds, including anthracycline, into toxic alcohols. In fact, several studies revealed that CBR could reduce anthracycline drugs to their C-13 hydroxy metabolites, resulting in the formation of cardiotoxic alcohol in the myocardium, which is thought to be the major contributor leading to AIC [24, 59]. At least one variant, *rs1056892*, in the *CBR3* gene was identified to be associated with an increased risk of AIC in pediatric cancer patients treated with anthracycline, and this finding has been replicated [14, 60, 61]. Furthermore, functional analysis of the *CBR3* enzyme in a mouse model confirmed the ability of this enzyme to metabolize anthracycline drugs such as doxorubicin [62].

*GSTM1* encodes glutathione S-transferase M1, a member of the GST family of enzymes that catalyze the detoxification of many carcinogens, drugs including anthracycline, and other toxins. The GSTM1 protein also serves as a free radical scavenger that reduces the oxidative damage generated by toxic compounds such as anthracycline. Thus, it makes sense that any variants interfering with the expression level and/or functionality of the GSTM1 enzyme will thus increase the risk of AIC. Indeed, patients with the *GSTM1* null genotype, which results in reduced expression of GSTM1 enzyme activity, as evidenced from hiPSC-CMs derived from patients, showed an ~2.7-fold greater odds ratio of developing clinically diagnostic AIC [63].

*UGT1A6* encodes UDP-glucuronosyltransferase family 1 member A6, an enzyme involved in the detoxification glucuronidation pathway that transforms lipophilic molecules and drugs, including anthracycline, into water-soluble, excretable metabolites. Thus, the UGT1A6 protein is an important player in anthracycline drug clearance. The *rs17863783* variant has been identified to be associated with an increased risk of AIC in pediatric cancer patients treated with anthracycline [45, 57]. Because the association of this variant with an increased risk of AIC was well replicated in three independent patient cohorts, pharmacogenomic testing is further recommended for pediatric cancer patients who receive anthracycline chemotherapy [64].

### 3.3. Oxidative Stress Generation

One of the prevailing mechanisms of AIC involves the generation of oxidative stress. It is well studied that biochemical transformation of anthracyclines leads to the generation of reactive oxygen species (ROS). As cardiomyocytes contain very low levels of catalase (CAT) and selenium-dependent GSH-peroxidase-1 (GSH-PX1), two antioxidant enzymes capable of detoxifying activated oxygen [65], they in particular are extremely sensitive to ROS-induced damage compared to other cell types. This can be partially explained by the fact that the heart has a high demand for the metabolism of oxidative substrates to generate pump energy from a large number of mitochondria.

Nicotinamide adenine dinucleotide phosphate (NADPH) oxidase is an enzyme complex that is highly expressed in cardiomyocyte mitochondria. NADPH oxidase catalyzes the production of superoxide from oxygen and NADPH, thus serving as a major source of ROS generation in the myocardium and playing a key role in the altered risk of AIC. In line with this hypothesis, NADPH oxidase knockout mice exhibited a reduced risk of AIC in a mouse doxorubicin-induced cardiomyopathy and heart failure model [66]. Several genetic variants in other genes, including *CYBA*, *RAC2*, and *NCF4*, that encode subunits of the NADPH oxidase complex have been identified to be associated with an increased and/or decreased risk of AIC. The *CYBA* gene encodes the light chain (also known as alpha subunit) of cytochrome b-245, which is a component of the NADPH oxidase enzyme complex. Together with the *CYBB* gene that encodes the X-linked heavy chain (also known as beta subunit) of cytochrome, the *CYBA* gene is required for NADPH oxidase to function. The missense variant rs4673 in the *CYBA* gene was found to both increase and decrease the risk of AIC in different cohorts of cancer patients [51, 67]. The rs13058338 variant in the *RAC2* gene, which encodes a small regulator subunit of Rho GTPase that regulates the activation of NADPH oxidase, has also been identified to be associated with an increased risk of AIC [68]. In addition, *rs183112* in the neutrophil cytosolic factor 4 (NCF4) gene, which encodes the p40-phox subunit of NADPH oxidase, has been reported to be associated with an ~2.5- to 5-fold increased risk of AIC [69].

The nitric oxide synthase 3 (*NOS3*) gene encodes an enzyme that catalyzes the production of nitric oxide (NO). The *NOS3* gene is mostly expressed in endothelial tissue, where it mostly regulates the generation of NO in the circulatory system and heart. It was suggested that anthracycline could induce NOS expression and facilitate NOS production in the heart, which can ultimately promote anthracycline redox cycling to produce elevated ROS [70]. The variant *rs1799983* in the *NOS3* gene was identified to significantly reduce the risk of AIC in different pediatric cancer patient cohorts [48, 71].

Hyaluronan is a glycosaminoglycan constituting key components of the extracellular matrix. Hyaluronan serves as a scaffold in tissue remodeling, and it was found to be able to reduce ROS-induced cardiac injury. A functional study in a cell culture system revealed that hyaluronan could promote cardiomyocyte survival during ROS damage in vitro [72]. Hyaluronan is mostly synthesized by hyaluronan synthase-3 (HAS3). The variant *rs2232228* in the *HAS3* gene, which is believed to reduce the ability of HAS3 to synthesize hyaluronan, was identified to be associated with an ~9-fold increased risk of developing AIC in a cohort of pediatric cancer survivors [73]. More convincingly, the association of the *rs2232228* variant with increased susceptibility to AIC was replicated in an independent patient cohort.

### 3.4. DNA Damage

The inhibition of Top2*β*-mediated DNA double-strand breaks, and DNA damage is one of the prevailing molecular bases underlying the pathogenesis of AIC. Retinoic acid receptor gamma (*RARG*) encodes a retinoic acid (RA) receptor that belongs to the nuclear hormone receptor family. This RA receptor acts as a ligand-dependent transcriptional regulator by binding to the retinoic acid response elements (RAREs) found in the promoter regions of target genes. The *RARG* gene is highly expressed in the heart, and *Top2β* is one of its target genes. In a GWAS performed in pediatric cancer patients treated with anthracycline, independent replication was observed in similarly treated cohorts of patients. Aminkeng et al. identified a nonsynonymous variant (*rs2229774*, pSer427Leu) in the *RARG* coding region that was associated with an ~5-fold increased risk of AIC [74]. This variant was functionally validated to activate the expression level of *Top2b* in an in vitro cell culture system. Further functional studies in the hiPSC-CM system showed that this variant could significantly induce ROS generation, DNA double-strand breaks, and cell death after anthracycline treatment. In another large-scale independent GWAS, the *rs28714259* variant in the *RARG* gene was identified and replicated in independent breast cancer patient cohorts to be associated with an increased risk of AIC, but with no functional data provided [75].

In contrast to human genetic approaches, our group took advantage of a zebrafish genetic approach and identified myocardial but not epicardial activation of retinoid x receptor alpha (*RXRA*), another key component of the RA signaling pathway, conferring AIC protection effects in this animal model. This study further identified two FDA-approved RXRA agonists, isotretinoin and bexarotene, that exerted cardioprotective effects dependent upon anthracycline treatment. This study thus provides the first in vivo genetic evidence supporting *RXRA* as a therapeutic target for AIC that can inform future studies on its translational potential [76].

### 3.5. Iron Homeostasis

Increased free iron load in the heart has been shown to potentiate AIC [25]. The high FE2+ (HFE) gene encodes an MHC class I-like protein that regulates the body's iron absorption and homeostasis [77]. An early functional study of the *Hfe* gene in a mouse animal model showed that *Hfe* deficiency led to iron accumulation in the heart concurrent with significantly increased susceptibility to doxorubicin-induced cardiotoxicity and heart failure [78]. In line with this in vivo functional evidence, genetic variants, including *rs1799945* and *rs1899562*, in the *HFE* gene that could potentially increase iron deposition in the heart were associated with an increased risk of AIC in both pediatric and breast cancer patients who received anthracycline chemotherapies [68, 79–81]. Mostly derived from mechanistic studies on iron homeostasis, the iron-chelator dexrazoxane, which can effectively inhibit the production of ROS, is the only FDA- and European Medicines Agency- (EMA-) approved drug for AIC treatment and prevention [82].

### 3.6. Sarcomere Dysfunction

The CUGBP Elav-Like Family Member 4 (*CELF4*) gene encodes an RNA binding protein involved in tissue-specific, developmentally regulated RNA processing. *TNNT2*, which encodes cardiac troponin T, is one of the prominent CELF4 binding target genes. Cardiac troponin T is one of the key components of the troponin protein complex that makes up part of the thin filament of the sarcomere structure. The CC genotype of the rs1786814 variant was demonstrated to be coexpressed with multiple *TNNT2* splice variants and thus associated with an ~10-fold increased risk of AIC in pediatric cancer survivors exposed to anthracycline doses of >300 mg/m^2^. Importantly, this association was further replicated in an independent patient cohort. Mechanistically, the modifying effect of the CC genotype in the *rs1786814* variant was speculated to occur through a pathway involving the expression of abnormally spliced *TNNT2* variants [83].

Titin, encoded by the *TTN* gene, serves as the major sarcomeric scaffold and has a regulatory function during cardiac contraction. *TTN* truncating variants (*TTNtvs*) are the most common causes of dilated cardiomyopathy (DCM), accounting for up to 25% of DCM cases. Based on evidence from targeted sequence studies in patient cohorts, two recent studies found that *TTNtvs* were also significantly associated with an increased risk of AIC in both pediatric and adult cancer survivors [84, 85]. Mechanistically, it remains to be investigated whether *TTNtvs* are a direct cause of an increased risk of AIC or just a secondary effect due to their known association with DCM and heart failure.

## 4. Conclusions and Perspectives

The goal of identifying genetic susceptibility and elucidating the underlying pathogenesis for AIC is for early prediction, intervention, and treatment. Our current understanding of AIC pathogenesis involves ROS generation, the inhibition of Top2*β* enzyme, autophagy dysregulation, and mitochondrial damage. Nevertheless, dexrazoxane remains as the solely mechanism-based drug approved by FDA for AIC treatment clinically. To date, more than 60 variants in approximately 40 affected genes, with different levels of association evidence, have been identified to be associated with AIC severity based on different levels of evidence, mostly through human genetic approaches such as candidate SNP assays and GWASs [4, 5]. Recommendations for genetic testing for some of the variants with the strongest evidence, such as *RARG rs2229774*, *SLC28A3 rs7853758*, and *UGT1A6 rs17863783*, have been proposed to be incorporated into a clinical AIC risk prediction model [64]. However, complicated by the large heterogeneity between different patient cohorts, in many cases, replication analysis for the majority of the other variants in independent studies failed to validate the initial findings. In most cases, functional validation for a large number of the variants identified thus far is needed to determine their exact causal effects, which represents a common challenge for many ongoing GWASs in AIC studies. Thus, association studies in human genetics alone have been underpowered. Currently, it is not feasible to accurately predict which patients will develop AIC more or less than others, and gene- and mechanism-based therapeutic strategies are still lacking.

Recently, hiPSC-CMs from patients have been shown to be able to recapitulate a patient's sensitivity to AIC, which provides a human relevant system to perform functional validation of variants of interest in vitro with reasonable throughput [86]. Despite of the issue of immaturity and in vitro nature of hiPSC-CMs which remain as two major challenges in their faithfully modeling human AIC [87], hiPSC-CMs might represent a novel platform for characterizing the genetic basis and pharmacogenomics of AIC. Findings resultant from hiPSC-CM studies can be further potentially used to guide the development of genetic screening tests [32, 88]. Notably, a recent study reported by Han et al. provides a remarkable example in utilizing the hiPSC-CM system to facilitate the discovery and subsequent elucidation of novel mechanistic underpinning of AIC [89]. This study revealed that the CirITCH (circular RNA ITCH (E3 ubiquitin-protein ligase)) mitigated AIC through acting as a sponge that sequestered the miR-330-5p, which shed light on the novel molecular mechanism of AIC. Overexpression of the CirITCH in both the in vitro hiPSC-CMs and in vivo mouse animal models protected against AIC. These findings thus can be potentially leveraged for future development of new pharmacologic intervention for AIC.

With the advent of *CRISPR/Cas9*, which enables us to efficiently edit every gene theoretically at the whole-animal level [90, 91], the zebrafish, with its vertebrate animal organism nature yet smaller size, lower cost, and higher throughput than rodent models, is emerging as a prolific in vivo animal model for identification of novel genetic factors and functional testing of a large number of variants of unknown significance (VUSs) in AIC studies. For example, we recently developed a forward mutagenesis screening approach in adult zebrafish for systematic discovery of genetic susceptibility to AIC. As a result, both known and novel genetic factors including *ANO5*, *DNAJB6*, *RXRA*, *SORBS2*, and *MAP7D1* were identified to modify the AIC disease progression [76, 92–94]. Particularly, detailed functional study on the *RXRA* gene revealed therapeutic benefit of endothelial cell-type specific activation of RXRA signaling on AIC [76]. Subsequently, two RXRA agonists, when administrated in the early but not the late AIC phase, were identified to exert AIC protection. More recently, through initial genetic studies in zebrafish animal models, we revealed dynamic regulation of autophagy in AIC [38]. We then carried out autophagy pathway targeted pharmacologic screening and identified two autophagy activators that exerted therapeutic effects in the late, but not early phase of AIC. While the conservation issue between zebrafish animal models and human needs to be cautioned for translational application, these research advances highlight the importance of stage-dependent treatment regimen that should be considered for future development of AIC therapeutics.

In summary, to effectively define the precise genotype-phenotype relationships for the vast putative genetic loci identified by human genetic approaches, a plausible future research strategy would be to integrate hiPSC-CMs and/or animal models such as zebrafish into human genetic studies for rapid functional validation and mechanistic investigation. With powerful drug screening platforms readily available in these models, targeted pharmacologic screening could be subsequently carried out for the top candidate variants/genes involved pathways. Thus, the complementary strength of human genetics and iPSC-CMs and zebrafish animal models is expected to systematically identify and elucidate AIC-associated gene loci that will likely improve our understanding of the pathogenesis of AIC mechanisms. The resultant findings can thus ultimately inform the incorporation of genetic testing and pharmacogenomic treatment for AIC into clinical practice.

## References

[1] Groarke J. D., Nohria A. (2015). Anthracycline cardiotoxicity. *Circulation*.

[2] Armenian S., Bhatia S. (2018). Predicting and preventing anthracycline-related cardiotoxicity. *American Society of Clinical Oncology Educational Book*.

[3] Robertson J., Barr R., Shulman L. N., Forte G. B., Magrini N. (2016). Essential medicines for cancer: WHO recommendations and national priorities. *Bulletin of the World Health Organization*.

[4] Bhatia S. (2020). Genetics of anthracycline cardiomyopathy in cancer survivors: JACC: cardiooncology state-of-the-art review. *Cardio Oncology*.

[5] Petrykey K., Andelfinger G. U., Laverdiere C., Sinnett D., Krajinovic M. (2020). Genetic factors in anthracycline-induced cardiotoxicity in patients treated for pediatric cancer. *Expert Opinion on Drug Metabolism & Toxicology*.

[6] Miller K. D., Siegel R. L., Lin C. C. (2016). Cancer treatment and survivorship statistics, 2016. *CA: a Cancer Journal for Clinicians*.

[7] Bansal N., Adams M. J., Ganatra S. (2019). Strategies to prevent anthracycline-induced cardiotoxicity in cancer survivors. *Cardiooncology*.

[8] Volkova M., Russell R. (2011). Anthracycline cardiotoxicity: prevalence, pathogenesis and treatment. *Current Cardiology Reviews*.

[9] Cardinale D., Colombo A., Bacchiani G. (2015). Early detection of anthracycline cardiotoxicity and improvement with heart failure therapy. *Circulation*.

[10] Cowgill J. A., Francis S. A., Sawyer D. B. (2019). Anthracycline and peripartum cardiomyopathies. *Circulation Research*.

[11] Curigliano G., Cardinale D., Dent S. (2016). Cardiotoxicity of anticancer treatments: epidemiology, detection, and management. *CA: a Cancer Journal for Clinicians*.

[12] Floyd J. D., Nguyen D. T., Lobins R. L., Bashir Q., Doll D. C., Perry M. C. (2005). Cardiotoxicity of cancer therapy. *Journal of Clinical Oncology*.

[13] Swain S. M., Whaley F. S., Ewer M. S. (2003). Congestive heart failure in patients treated with doxorubicin. *Cancer*.

[14] Blanco J. G., Sun C. L., Landier W. (2012). Anthracycline-related cardiomyopathy after childhood cancer: role of polymorphisms in carbonyl reductase genes--a report from the Children’s Oncology Group. *Journal of Clinical Oncology*.

[15] Fogarassy G., Fogarassyne Vathy A., Kovats T. (2020). Analysing the risk factors of doxorubicin-associated heart failure by a retrospective study of integrated, nation-wide databases. *Orvosi Hetilap*.

[16] Lotrionte M., Biondi-Zoccai G., Abbate A. (2013). Review and meta-analysis of incidence and clinical predictors of anthracycline cardiotoxicity. *The American Journal of Cardiology*.

[17] Qiu S., Zhou T., Qiu B. (2021). Risk factors for anthracycline-induced cardiotoxicity. *Frontiers in cardiovascular medicine*.

[18] Saleh Y., Abdelkarim O., Herzallah K., Abela G. S. (2021). Anthracycline-induced cardiotoxicity: mechanisms of action, incidence, risk factors, prevention, and treatment. *Heart Failure Reviews*.

[19] Zhang M., Yang H., Xu C., Jin F., Zheng A. (2022). Risk factors for anthracycline-induced cardiotoxicity in breast cancer treatment: a meta-analysis. *Frontiers in Oncology*.

[20] Ewer M. S., Ewer S. M. (2015). Cardiotoxicity of anticancer treatments. *Nature Reviews. Cardiology*.

[21] Berkman A. M., Hildebrandt M. A. T., Landstrom A. P. (2021). The genetic underpinnings of anthracycline-induced cardiomyopathy predisposition. *Clinical Genetics*.

[22] Norton N., Weil R. M., Advani P. P. (2021). Inter-individual variation and cardioprotection in anthracycline-induced heart failure. *Journal of Clinical Medicine*.

[23] Tripaydonis A., Conyers R., Elliott D. A. (2019). Pediatric anthracycline-induced cardiotoxicity: mechanisms, pharmacogenomics, and pluripotent stem-cell modeling. *Clinical Pharmacology and Therapeutics*.

[24] Minotti G., Menna P., Salvatorelli E., Cairo G., Gianni L. (2004). Anthracyclines: molecular advances and pharmacologic developments in antitumor activity and cardiotoxicity. *Pharmacological Reviews*.

[25] Link G., Tirosh R., Pinson A., Hershko C. (1996). Role of iron in the potentiation of anthracycline cardiotoxicity: identification of heart cell mitochondria as a major site of iron- anthracycline interaction. *The Journal of Laboratory and Clinical Medicine*.

[26] Sterba M., Popelova O., Vavrova A. (2013). Oxidative stress, redox signaling, and metal chelation in anthracycline cardiotoxicity and pharmacological cardioprotection. *Antioxidants & Redox Signaling*.

[27] Jones R. L. (2008). Utility of dexrazoxane for the reduction of anthracycline-induced cardiotoxicity. *Expert Review of Cardiovascular Therapy*.

[28] Simunek T., Sterba M., Popelova O., Adamcova M., Hrdina R., Gersl V. (2009). Anthracycline-induced cardiotoxicity: overview of studies examining the roles of oxidative stress and free cellular iron. *Pharmacological Reports*.

[29] Acar Z., Kale A., Turgut M. (2011). Efficiency of atorvastatin in the protection of anthracycline-induced cardiomyopathy. *Journal of the American College of Cardiology*.

[30] Avila M. S., Ayub-Ferreira S. M., de Barros Wanderley M. R. (2018). Carvedilol for prevention of chemotherapy-related cardiotoxicity: the CECCY trial. *Journal of the American College of Cardiology*.

[31] Kalam K., Marwick T. H. (2013). Role of cardioprotective therapy for prevention of cardiotoxicity with chemotherapy: a systematic review and meta-analysis. *European Journal of Cancer*.

[32] Tashakori Beheshti A., Mostafavi Toroghi H., Hosseini G., Zarifian A., Homaei Shandiz F., Fazlinezhad A. (2016). Carvedilol administration can prevent doxorubicin-induced cardiotoxicity: a double-blind randomized trial. *Cardiology*.

[33] Vejpongsa P., Yeh E. T. (2014). Prevention of anthracycline-induced cardiotoxicity: challenges and opportunities. *Journal of the American College of Cardiology*.

[34] Vejpongsa P., Yeh E. T. (2014). Topoisomerase 2*β*: a promising molecular target for primary prevention of anthracycline-induced cardiotoxicity. *Clinical Pharmacology and Therapeutics*.

[35] Zhang S., Liu X., Bawa-Khalfe T. (2012). Identification of the molecular basis of doxorubicin-induced cardiotoxicity. *Nature Medicine*.

[36] Li D. L., Wang Z. V., Ding G. (2016). Doxorubicin blocks cardiomyocyte autophagic flux by inhibiting lysosome acidification. *Circulation*.

[37] Li M., Sala V., De Santis M. C. (2018). Phosphoinositide 3-kinase gamma inhibition protects from anthracycline cardiotoxicity and reduces tumor growth. *Circulation*.

[38] Wang Y., Lu X., Wang X. (2021). <i>atg7</i>-Based autophagy activation reverses doxorubicin-induced cardiotoxicity. *Circulation research*.

[39] Huang J., Wu R., Chen L., Yang Z., Yan D., Li M. (2022). Understanding anthracycline cardiotoxicity from mitochondrial aspect. *Frontiers in Pharmacology*.

[40] Murabito A., Hirsch E., Ghigo A. (2020). Mechanisms of anthracycline-induced cardiotoxicity: is mitochondrial dysfunction the answer?. *Frontiers in cardiovascular medicine*.

[41] Wallace K. B., Sardao V. A., Oliveira P. J. (2020). Mitochondrial determinants of doxorubicin-induced cardiomyopathy. *Circulation Research*.

[42] Tadokoro T., Ikeda M., Ide T. (2020). Mitochondria-dependent ferroptosis plays a pivotal role in doxorubicin cardiotoxicity. *JCI Insight*.

[43] Linschoten M., Teske A. J., Cramer M. J., van der Wall E., Asselbergs F. W. (2018). Chemotherapy-related cardiac dysfunction: a systematic review of genetic variants modulating individual risk. *Circulation: Genomic and Precision Medicine*.

[44] Visscher H., Rassekh S. R., Sandor G. S. (2015). Genetic variants in SLC22A17 and SLC22A7 are associated with anthracycline-induced cardiotoxicity in children. *Pharmacogenomics*.

[45] Visscher H., Ross C. J., Rassekh S. R. (2012). Pharmacogenomic prediction of anthracycline-induced cardiotoxicity in children. *Journal of clinical oncology*.

[46] Cascorbi I. (2006). Role of pharmacogenetics of ATP-binding cassette transporters in the pharmacokinetics of drugs. *Pharmacology & Therapeutics*.

[47] Couture L., Nash J. A., Turgeon J. (2006). The ATP-binding cassette transporters and their implication in drug disposition: a special look at the heart. *Pharmacological Reviews*.

[48] Krajinovic M., Elbared J., Drouin S. (2016). Polymorphisms of ABCC5 and NOS3 genes influence doxorubicin cardiotoxicity in survivors of childhood acute lymphoblastic leukemia. *The Pharmacogenomics Journal*.

[49] Pratt S., Shepard R. L., Kandasamy R. A., Johnston P. A., Perry W., Dantzig A. H. (2005). The multidrug resistance protein 5 (ABCC5) confers resistance to 5-fluorouracil and transports its monophosphorylated metabolites. *Molecular Cancer Therapeutics*.

[50] Semsei A. F., Erdelyi D. J., Ungvari I. (2012). ABCC1 polymorphisms in anthracycline-induced cardiotoxicity in childhood acute lymphoblastic leukaemia. *Cell Biology International*.

[51] Wojnowski L., Kulle B., Schirmer M. (2005). NAD (P) H oxidase and multidrug resistance protein genetic polymorphisms are associated with doxorubicin-induced cardiotoxicity. *Circulation*.

[52] Huang J. F., Wen C. J., Zhao G. Z. (2018). Overexpression of ABCB4 contributes to acquired doxorubicin resistance in breast cancer cells in vitro. *Cancer Chemotherapy and Pharmacology*.

[53] Leong S. L., Chaiyakunapruk N., Lee S. W. (2017). Candidate gene association studies of anthracycline-induced cardiotoxicity: a systematic review and meta-analysis. *Scientific Reports*.

[54] Jungsuwadee P., Nithipongvanitch R., Chen Y. (2009). Mrp1 localization and function in cardiac mitochondria after doxorubicin. *Molecular Pharmacology*.

[55] Ichikawa Y., Ghanefar M., Bayeva M. (2014). Cardiotoxicity of doxorubicin is mediated through mitochondrial iron accumulation. *The Journal of Clinical Investigation*.

[56] Menon A. V., Kim J. (2022). Iron promotes cardiac doxorubicin retention and toxicity through downregulation of the mitochondrial exporter ABCB8. *Frontiers in Pharmacology*.

[57] Visscher H., Ross C. J., Rassekh S. R. (2013). Validation of variants in SLC28A3 and UGT1A6 as genetic markers predictive of anthracycline-induced cardiotoxicity in children. *Pediatric Blood & Cancer*.

[58] Sagi J. C., Egyed B., Kelemen A. (2018). Possible roles of genetic variations in chemotherapy related cardiotoxicity in pediatric acute lymphoblastic leukemia and osteosarcoma. *BMC Cancer*.

[59] Sagi J. C., Kutszegi N., Kelemen A. (2016). Pharmacogenetics of anthracyclines. *Pharmacogenomics*.

[60] Serie D. J., Crook J. E., Necela B. M. (2017). Genome-wide association study of cardiotoxicity in the NCCTG N9831 (alliance) adjuvant trastuzumab trial. *Pharmacogenetics and Genomics*.

[61] Blanco J. G., Leisenring W. M., Gonzalez-Covarrubias V. M. (2008). Genetic polymorphisms in the carbonyl reductase 3 gene CBR3 and the NAD (P)H: quinone oxidoreductase 1 gene NQO1 in patients who developed anthracycline-related congestive heart failure after childhood cancer. *Cancer*.

[62] Schaupp C. M., White C. C., Merrill G. F., Kavanagh T. J. (2015). Metabolism of doxorubicin to the cardiotoxic metabolite doxorubicinol is increased in a mouse model of chronic glutathione deficiency: a potential role for carbonyl reductase 3. *Chemico-Biological Interactions*.

[63] Singh P., Wang X., Hageman L. (2020). Association of GSTM1 null variant with anthracycline-related cardiomyopathy after childhood cancer-a Children’s Oncology Group ALTE03N1 report. *Cancer*.

[64] Aminkeng F., Ross C. J., Rassekh S. R. (2016). Recommendations for genetic testing to reduce the incidence of anthracycline-induced cardiotoxicity. *British Journal of Clinical Pharmacology*.

[65] Doroshow J. H., Locker G. Y., Myers C. E. (1980). Enzymatic defenses of the mouse heart against reactive oxygen metabolites: alterations produced by doxorubicin. *The Journal of Clinical Investigation*.

[66] Zhao Y., McLaughlin D., Robinson E. (2010). Nox2 NADPH oxidase promotes pathologic cardiac remodeling associated with doxorubicin chemotherapy. *Cancer Research*.

[67] Megias-Vericat J. E., Montesinos P., Herrero M. J. (2018). Impact of NADPH oxidase functional polymorphisms in acute myeloid leukemia induction chemotherapy. *The Pharmacogenomics Journal*.

[68] Armenian S. H., Ding Y., Mills G. (2013). Genetic susceptibility to anthracycline-related congestive heart failure in survivors of haematopoietic cell transplantation. *British Journal of Haematology*.

[69] Cascales A., Pastor-Quirante F., Sanchez-Vega B. (2013). Association of anthracycline-related cardiac histological lesions with NADPH oxidase functional polymorphisms. *The Oncologist*.

[70] Fogli S., Nieri P., Breschi M. C. (2004). The role of nitric oxide in anthracycline toxicity and prospects for pharmacologic prevention of cardiac damage. *The FASEB Journal*.

[71] McOwan T. N., Craig L. A., Tripdayonis A. (2020). Evaluating anthracycline cardiotoxicity associated single nucleotide polymorphisms in a paediatric cohort with early onset cardiomyopathy. *Cardiooncology*.

[72] Law C. H., Li J. M., Chou H. C., Chen Y. H., Chan H. L. (2013). Hyaluronic acid-dependent protection in H9C2 cardiomyocytes: a cell model of heart ischemia-reperfusion injury and treatment. *Toxicology*.

[73] Wang X., Liu W., Sun C. L. (2014). Hyaluronan synthase 3 variant and anthracycline-related cardiomyopathy: a report from the Children’s Oncology Group. *Journal of Clinical Oncology*.

[74] Aminkeng F., Bhavsar A. P., Visscher H. (2015). A coding variant in RARG confers susceptibility to anthracycline-induced cardiotoxicity in childhood cancer. *Nature genetics*.

[75] Schneider B. P., Shen F., Gardner L. (2017). Genome-wide association study for anthracycline-induced congestive heart failure. *Clinical Cancer Research*.

[76] Ma X., Zhu P., Ding Y. (2020). Retinoid X receptor alpha is a spatiotemporally predominant therapeutic target for anthracycline-induced cardiotoxicity. *Science advances*.

[77] Barton J. C., Edwards C. Q., Acton R. T. (2015). HFE gene: structure, function, mutations, and associated iron abnormalities. *Gene*.

[78] Miranda C. J., Makui H., Soares R. J. (2003). Hfe deficiency increases susceptibility to cardiotoxicity and exacerbates changes in iron metabolism induced by doxorubicin. *Blood*.

[79] Lipshultz S. E., Lipsitz S. R., Kutok J. L. (2013). Impact of hemochromatosis gene mutations on cardiac status in doxorubicin-treated survivors of childhood high-risk leukemia. *Cancer*.

[80] Vaitiekus D., Muckiene G., Vaitiekiene A. (2021). HFE gene variants’ impact on anthracycline-based chemotherapy-induced subclinical cardiotoxicity. *Cardiovascular Toxicology*.

[81] Armenian S. H., Lacchetti C., Barac A. (2017). Prevention and monitoring of cardiac dysfunction in survivors of adult cancers: American Society of Clinical Oncology clinical practice guideline. *Journal of Clinical Oncology*.

[82] Cai F., Luis M. A. F., Lin X. (2019). Anthracycline-induced cardiotoxicity in the chemotherapy treatment of breast cancer: preventive strategies and treatment. *Molecular and clinical oncology*.

[83] Wang X., Sun C. L., Quinones-Lombrana A. (2016). CELF4 variant and anthracycline-related cardiomyopathy: a Children’s Oncology Group genome-wide association study. *Journal of Clinical Oncology*.

[84] Garcia-Pavia P., Kim Y., Restrepo-Cordoba M. A. (2019). Genetic variants associated with cancer therapy-induced cardiomyopathy. *Circulation*.

[85] Linschoten M., Teske A. J., Baas A. F. (2017). Truncating titin (TTN) variants in chemotherapy-induced cardiomyopathy. *Journal of Cardiac Failure*.

[86] Burridge P. W., Li Y. F., Matsa E. (2016). Human induced pluripotent stem cell-derived cardiomyocytes recapitulate the predilection of breast cancer patients to doxorubicin-induced cardiotoxicity. *Nature Medicine*.

[87] Koivumaki J. T., Naumenko N., Tuomainen T. (2018). Structural immaturity of human iPSC-derived cardiomyocytes: in silico investigation of effects on function and disease modeling. *Frontiers in Physiology*.

[88] Lo Sardo V., Kamp T. J. (2022). Preventing anthracycline-induced cardiotoxicity using functional genomics and human-induced pluripotent stem cell-derived cardiomyocytes. *Circulation*.

[89] Han D., Wang Y., Wang Y. (2020). The tumor-suppressive human circular RNA CircITCH sponges miR-330-5p to ameliorate doxorubicin-induced cardiotoxicity through upregulating SIRT6, survivin, and SERCA2a. *Circulation Research*.

[90] Albadri S., De Santis F., Di Donato V., Del Bene F., Jaenisch R., Zhang F., Gage F. (2017). CRISPR/Cas9-mediated knockin and knockout in zebrafish. *Genome Editing in Neurosciences*.

[91] Cornet C., Di Donato V., Terriente J. (2018). Combining zebrafish and CRISPR/Cas9: toward a more efficient drug discovery pipeline. *Frontiers in Pharmacology*.

[92] Ding Y., Long P. A., Bos J. M. (2016). A modifier screen identifies DNAJB6 as a cardiomyopathy susceptibility gene. *JCI Insight*.

[93] Ding Y., Yang J., Chen P. (2020). Knockout of SORBS2 protein disrupts the structural integrity of intercalated disc and manifests features of arrhythmogenic cardiomyopathy. *Journal of the American Heart Association*.

[94] Li L. P., Zhong J., Li M. H. (2021). Disruption of MAP7D1 gene function increases the risk of doxorubicin-induced cardiomyopathy and heart failure. *BioMed Research International*.

